# Synergistic regulation mechanism of nitrogen and potassium coupling on sugarcane yield and soil quality in karst areas

**DOI:** 10.3389/fpls.2025.1614682

**Published:** 2025-09-18

**Authors:** Ning Zhong, Qiuliang Cai, Jinsheng Huang

**Affiliations:** ^1^ Fujian Province Key Laboratory of Modern Analytical Science and Separation Technology, College of Chemistry, Chemical Engineering & Environmental Science, Minnan Normal University, Zhangzhou, China; ^2^ Guangxi Key Laboratory of Biology for Mango, Agriculture and Food Engineering College, Baise University, Industrial College of Subtropical Characteristic Agriculture, Baise, Guangxi, China; ^3^ Agricultural Resources and Environmental Research Institute, Guangxi Academy of Agricultural Sciences, Nanning, Guangxi, China

**Keywords:** nitrogen potassium coupling, sugarcane yield, soil enzyme activity, ecological chemometrics, karst area

## Abstract

To address the conflict between soil degradation and sugarcane productivity in South China karst regions, this study investigated the coordinated regulatory effects of fertilization practices on sugarcane yield, soil enzymatic activities, and ecological stoichiometry through nitrogen-potassium coupling experiments, establishing a theoretical framework for precision nutrient management. A field experiment evaluated the soil enzymatic activities, nutrient concentrations, ecological stoichiometry indices, and sugarcane productivity across eight nitrogen-potassium application ratios (N_0_K_0_ to N_3_K_2_). The direct and indirect effects of fertilization measures were quantified using structural equation model (SEM), grey relational analysis and path analysis. The results showed that the high nitrogen and high potassium treatment (N_3_K_2_) achieved maximum sugarcane yield (91.05 t ha^-1^), representing a 55.25% increase over the control, but leading to soil acidification (pH 4.57). Optimal nitrogen use efficiency (38.47%) occurred under the low nitrogen and high potassium treatment (N_1_K_2_), exceeding conventional fertilization by 21.41%. Path analysis revealed that urease exerted the strongest direct yield effect (1.977), while total potassium demonstrated the highest grey correlation degree (0.911). The medium nitrogen and high potassium treatment (N_2_K_2_) exhibited optimal soil fertility (IFI = 0.57) with significantly higher total base ions (0.48 Cmol kg^-1^) compared to other treatments. The ecological stoichiometric characteristics showed that the soil in the study area was in the state of nitrogen limitation (nitrogen-phosphorus ratio = 1.89) and phosphorus efficiency (carbon-nitrogen ratio = 9.56-23.25). Structural equation modeling revealed nutrient cycling influenced sugarcane yield through enzymatic regulation (total effect = 0.702). This study established an acid soil ternary regulation framework integrating enzyme activity, nutrient cycling, and stoichiometric balance, while recommending the optimization scheme of nitrogen-potassium ratio (N:K_2_O ≈ 1:1.5) for southern sugarcane cultivation. The ammonium nitrogen activation coefficient (> 1.5%) and carbon-phosphorus ratio (< 25%) were identified as pivotal fertility monitoring parameters, offering theoretical foundations for precision fertilization and sustainable soil management in karst areas.

## Introduction

Contemporary global agriculture confronts simultaneous soil degradation and food security challenges (Atanda et al., 2025). As an important sugarcane production area in southern China, the karst areas have prominent problems such as soil acidification and nutrient loss caused by ecological vulnerability and high-intensity agricultural development (Gao et al., 2024). Sugarcane (*Saccharum officinarum L.*), characterized by high biomass production, demonstrates yield formation processes fundamentally dependent on the supply of nitrogen and potassium nutrients (Bhatt et al., 2024). However, conventional nitrogen fertilizer over-application results in low utilization efficiency (20%-35%) (Khanam et al., 2025), while improper potassium fertilization exacerbates the risk of soil degradation (Roeva et al., 2023). Within agricultural green transformation context, elucidating nitrogen-potassium coupling mechanisms for optimizing sugarcane productivity and soil quality carries critical implications for sustainable karst agroecosystem management.

Nitrogen, as an essential constituent of chlorophyll and proteins, critically influences sugarcane growth, with deficiency causing a 20%-30% reduction in tiller number (Bhatt et al., 2024), while excess levels impair sucrose synthesis (Hisbani et al., 2025). Potassium mediates osmotic regulation and mechanical strength, in which deficiency elevates stem breakage incidence by 40% (Roeva et al., 2023) while facilitating photosynthate translocation to the stem (Abayomi, 1987). Nitrogen fertilizer input can exacerbate soil acidification through proton release (2.8 mol H^+^ can be produced for every 1 kg of urea applied, Liu et al., 2024), while potassium ions in potassium fertilizer can replace calcium and magnesium ions on soil colloids, affecting base saturation (Batjes, 1996). Extensive research has documented the nitrogen-potassium ratio effects on crop productivity. Potassium fertilization enhances sugarcane biomass (Hisbani et al., 2025; Tessier and Raynal, 2003), though excessive levels compromise nitrogen uptake, while synergistic nitrogen-potassium interactions boost yield through photosynthetic regulation (Abayomi, 1987). However, current knowledge remains limited to isolated nutrient responses and short-term observations, lacking integrated assessments of soil enzymatic activity, nutrient cycling dynamics, and ecological stoichiometric balances. As a biocatalyst for nutrient transformation, soil enzyme activity changes directly affect nitrogen mineralization and phosphorus activation (Hu et al., 2025; Gao et al., 2024). Ecological stoichiometry research has shown that carbon-nitrogen ratio (C: N), nitrogen-phosphorus ratio (N: P) and other indicators effectively indicate soil nutrient limitation types (Koerselman and Meuleman, 1996), though such applications remain unexplored in sugarcane fertilization research. In addition, traditional approaches predominantly employ basic correlation analyses, failing to quantitatively establish causal relationships among fertilization regimes, enzyme activities, nutrient cycling processes, and ultimate yield outcomes (Sun et al., 2025).

Addressing these research deficiencies, this investigation focuses on Baise karst sugarcane region in Guangxi, employing field positioning experiments and interdisciplinary approaches to determine the optimal ratio of nitrogen-potassium coupling for sugarcane yield enhancement and elucidate underlying physiological mechanisms. It will clarify the dynamic response characteristics of soil enzyme activity and nutrient cycles, gain insight into the collaborative regulation among enzyme activities, nutrient cycling, and ecological stoichiometry in acid soil. These findings will facilitate the development of fertilization strategies balancing productivity and sustainability, ultimately contributing to improved nutrient resource utilization in southern sugarcane cultivation systems and supporting agricultural sustainability transitions.

## 2 Materials and methods

### 2.1 Test materials and test scheme

The study area is located in the sugarcane planting area of Tianyang District, Baise City, Guangxi Zhuang Autonomous Region (106° 22 ′ 14 ″ E~107° 8 ′ 32 ″, 23° 28 ′ 20 ″~24° 6 ′ 55 ″ N). The study area experiences a subtropical monsoon climate characterized by an annual average temperature of 21.9 °C and an annual precipitation of 1156 mm. Experimental soils classified as yellow brown exhibited the following surface layer (0–20 cm) properties: total nitrogen (0.47 g kg^-1^), total phosphorus content (0.25 g kg^-1^), total potassium content (6.67 g kg^-1^), organic matter content (16.74 g kg^-1^), and pH (5.74). The investigated sugarcane cultivar Guitang 46 displays medium-large stem morphology with superior ratooning ability, coupled with high productivity and sucrose accumulation potential. The growth period of sugarcane is from February to December, the planting row spacing is 0.9~1.2 meters, and the theoretical yield is about 90 tons per ha.

Experimental procedures involved selecting the upper stem portions from newly cultivated sugarcane of Guitang 46 in February 2022. These stems were precision-sectioned into uniform double-bud segments using surgical blades, with only intact segments employed for planting. The experimental layout comprised 208 buds in total arranged in 52-bud rows under randomized complete block design. Eight nitrogen-potassium ratio treatments were implemented, each replicated three times. The plot area was 31.2 m^2^ (6.5 m × 4.8 m), and nitrogen fertilizer (urea, containing N 46%), potassium fertilizer (potassium chloride, containing K_2_O 60%) and phosphorus fertilizer (calcium superphosphate, containing P_2_O_5_ 12%) were applied in a ditch at one time. The specific treatment and fertilization statuses are shown in **Table 1**.

**Table 1:** The specific treatment and fertilization status.

Treatment	Abbreviation	Urea (kg)	Potassium chloride (kg)	Calcium superphosphate (kg)
no fertilizer control	N_0_K_0_	0	0	0
conventional fertilization	NrKr	2.28	1.40	3.13
low nitrogen and medium potassium	N_1_K_1_	2.25	2.50	3.13
medium nitrogen and medium potassium	N_2_K_1_	3.50	2.50	3.13
high nitrogen and medium potassium	N_3_K_1_	4.50	2.50	3.13
low nitrogen and high potassium	N_1_K_2_	2.25	3.50	3.13
medium nitrogen and high potassium	N_2_K_2_	3.50	3.50	3.13
high nitrogen and high potassium	N_3_K_2_	4.50	2.50	3.13

### 2.2 Sample collection and processing

Soil samples: During the December harvest period, topsoil (0–20 cm depth) was collected from each plot using a five-point sampling method, with five soil cores were homogenized per plot. Following natural air-drying and mechanical grinding, samples were fractionated through a 20-mesh sieve (enzyme activity analysis) and an 80-mesh sieve (physicochemical characterization).

Plant samples: Two representative sugarcane plants per plot were separated into stems and leaves for fresh weight measurement. Samples underwent an initial 105°C desiccation for 30 minutes followed by 65°C drying to constant mass, then ground and sieved through 20-mesh for subsequent analysis.

### 2.3 Determination of soil samples

#### 2.3.1 Determination of soil nutrient properties

Key soil fertility indicators were identified as monitoring parameters for nutrient assessment. Analytical procedures followed standard soil agrochemical analysis (Bao, 2000): organic matter quantification via potassium dichromate titration, total nitrogen measurement employed the Kjeldahl method, and soil pH determination through potentiometric analysis. Total phosphorus content employed sulfuric-perchloric acid digestion, while ammonium nitrogen content utilized Nessler reagent spectrophotometry. Available phosphorus was measured via molybdenum-antimony anti-colorimetry (Olsen et al., 1954), with nitrate nitrogen was determined by UV spectrophotometry.

#### 2.3.2 Determination of soil enzyme activity

Soil enzyme activities were quantified using standardized colorimetric methods: cellulase and invertase via 3,5-dinitrosalicylic acid colorimetry (Guan, 1986), urease through phenol-sodium hypochlorite colorimetry (Tabatabai, 1994), and acid phosphatase employing *p*-nitrophenyl phosphate sodium colorimetry (Tabatabai, 1994). Catalase activity was measured by potassium permanganate titration (Guan, 1986).

### 2.4 Data processing

#### 2.4.1 Nitrogen fertilizer use efficiency

Nitrogen use efficiency (%) = (nitrogen absorption of crops in nitrogen application area - nitrogen absorption of crops in nitrogen free area)/nitrogen application amount (pure amount) × 100%.

#### 2.4.2 Evaluation of soil fertility

Nine key indicators (total nitrogen, total phosphorus, total potassium, ammonium nitrogen, nitrate nitrogen, available phosphorus, available potassium, organic matter, pH) were weighted using the integrated fuzzy index (IFI) method, incorporating parabolic/S-type membership functions and factor analysis (Zhao, 2023). The membership turning point values were presented in **Table 2**.

**Table 2:** Values of membership turning point.

IND	Inflection point			
	X1	X2	X3	X4
pH	4.5	6.5	7.5	8.5
Organic matter	10	30		
Total nitrogen	0.75	2		
Total phosphorus	0.4	1		
Total potassium	5	25		
Alkali-hydrolyzable nitrogen	60	180		
Effective phosphorus	3	20		
Quick effect potassium	40	150		

#### 2.4.3 Statistical analysis

Data processing utilized Excel 2023, while statistical analysis employed SPSS 26.0 with one-way ANOVA (nitrogen-potassium treatment combination as fixed factor at 8 levels, block as random effect with 3 replicates) followed by Duncan test at *P* < 0.05 significance level. Data visualization was conducted using Origin 2022. Structural equation modeling (SEM) and redundancy analysis (RDA) were performed using SmartPLS 4.0 and Canoco 5.0, respectively.

## 3 Results and analysis

### 3.1 Effects of different treatments on soil nutrient properties, and enzyme activity of sugarcane

**Figure 1:**
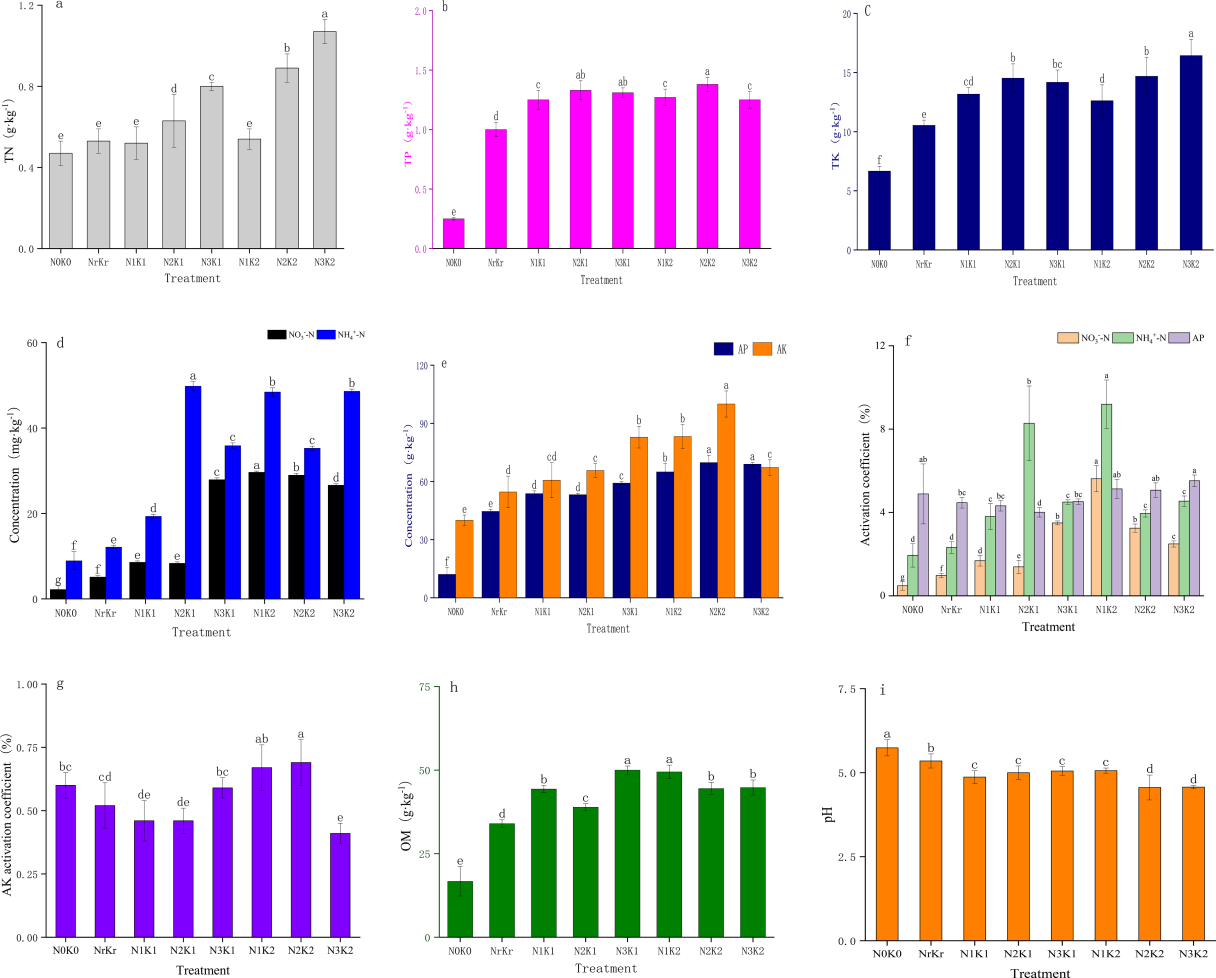
Effect of different treatments on soil fertility (mean ± SD, n = 3): total nitrogen **(a)**; total phosphorus **(b)**; total potassium **(c)**; Nitrate nitrogen and ammonium nitrogen **(d)**; available phosphorus and, available potassium **(e)**; Activating coefficient of Nitrate nitrogen, ammonium nitrogen and available phosphorus **(f)**; Activating coefficient of available potassium; Organic matter **(f)**; pH **(i)**; Different lowercase letters indicate significant differences among different fertilization treatments (*P* < 0.05), the same below.

Soil total nitrogen content exhibited a significant positive correlation with nitrogen fertilizer application rates (**Figure 1a**). The high nitrogen and high potassium treatment (N_3_K_2_) achieved the maximum nitrogen accumulation (1.07 g/kg), representing a 130% increase compared to the control (*P* < 0.01), while concurrently reducing soil pH to 4.57 (**Figure 1i**). The content of ammonium nitrogen reached the peak (49.81 mg kg^-1^) in the medium nitrogen and medium potassium treatment (N_2_K_1_), while the content of nitrate nitrogen was the highest (29.65 mg kg^-1^) in the low nitrogen and high potassium treatment (N_1_K_2_), indicating that appropriate nitrogen application promoted nitrate nitrogen accumulation (**Figure 1d**). Phosphorus fractions demonstrated minimal variation across treatments, except for total phosphorus, which significantly increased to 1.38 g kg^-1^ under high potassium application (N_2_K_2_) (**Figures 1b, e**). The contents of total potassium and available potassium exhibited dose-dependent responses, with N_3_K_2_ treatment achieving the maximum total potassium accumulation (16.44 g kg^-1^), representing a 147% increase relative to the control (**Figures 1c, e**). Soil organic matter peaked at 49.99 g kg^-1^ in the high nitrogen and medium potassium treatment (**Figure 1h**). Nitrogen activation analysis revealed significant negative correlations between application rates and activation coefficients for both ammonium nitrogen and nitrate nitrogen (*P* < 0.05). The high nitrogen treatment (N_3_K_2_) exhibited minimal ammonium nitrogen activation (4.54%) (**Figures 1f, g**), indicating nitrogen transformation inhibition under high nitrogen input. The maximum phosphorus activation (5.52%) occurred in the high nitrogen and high potassium treatment. **Table 3** data indicated optimal nitrogen use efficiency (38.47%) under low nitrogen-high potassium conditions, representing a 18.41% increase over conventional fertilization. Excessive nitrogen application (N_3_K_1_) reduced a nitrogen use efficiency to 22.34%, indicating that high nitrogen input reduced the nutrient use efficiency.

**Table 3:** Effects on nitrogen fertilizer utilization under different treatments.

IND	1 (N_0_K_0_)	2 (N_r_K_r_)	3 (N_1_K_1_)	4 (N_2_K_1_)	5 (N_3_K_1_)	6 (N_1_K_2_)	7 (N_2_K_2_)	8 (N_3_K_2_)
YD (t/ha)	58.65 ± 12.15	68.85 ± 6.75	68.55 ± 18.60	77.25 ± 9.45	90.90 ± 11.10	71.85 ± 16.80	82.35 ± 10.20	91.05 ± 16.95
NUE (%)		17.06	17.70	19.56	22.34	38.47	27.07	24.95

**Figure 2:**
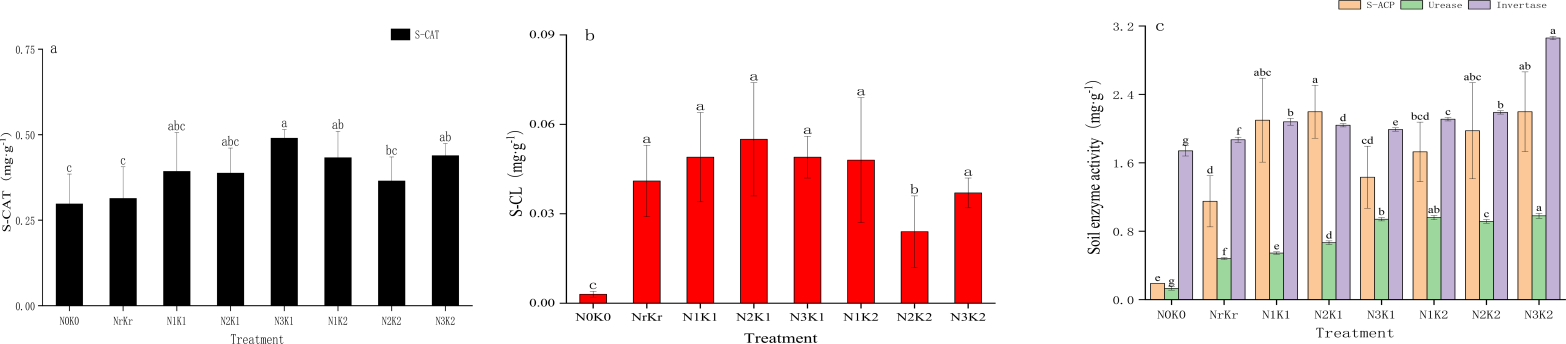
Effects of the different treatments on enzyme activities (mean ± SD, n = 3): catalase activity **(a)**; cellulase activity **(b)**; acid phosphatase activity, urease activity, and invertase activity **(c)**.

**Figure 3:**
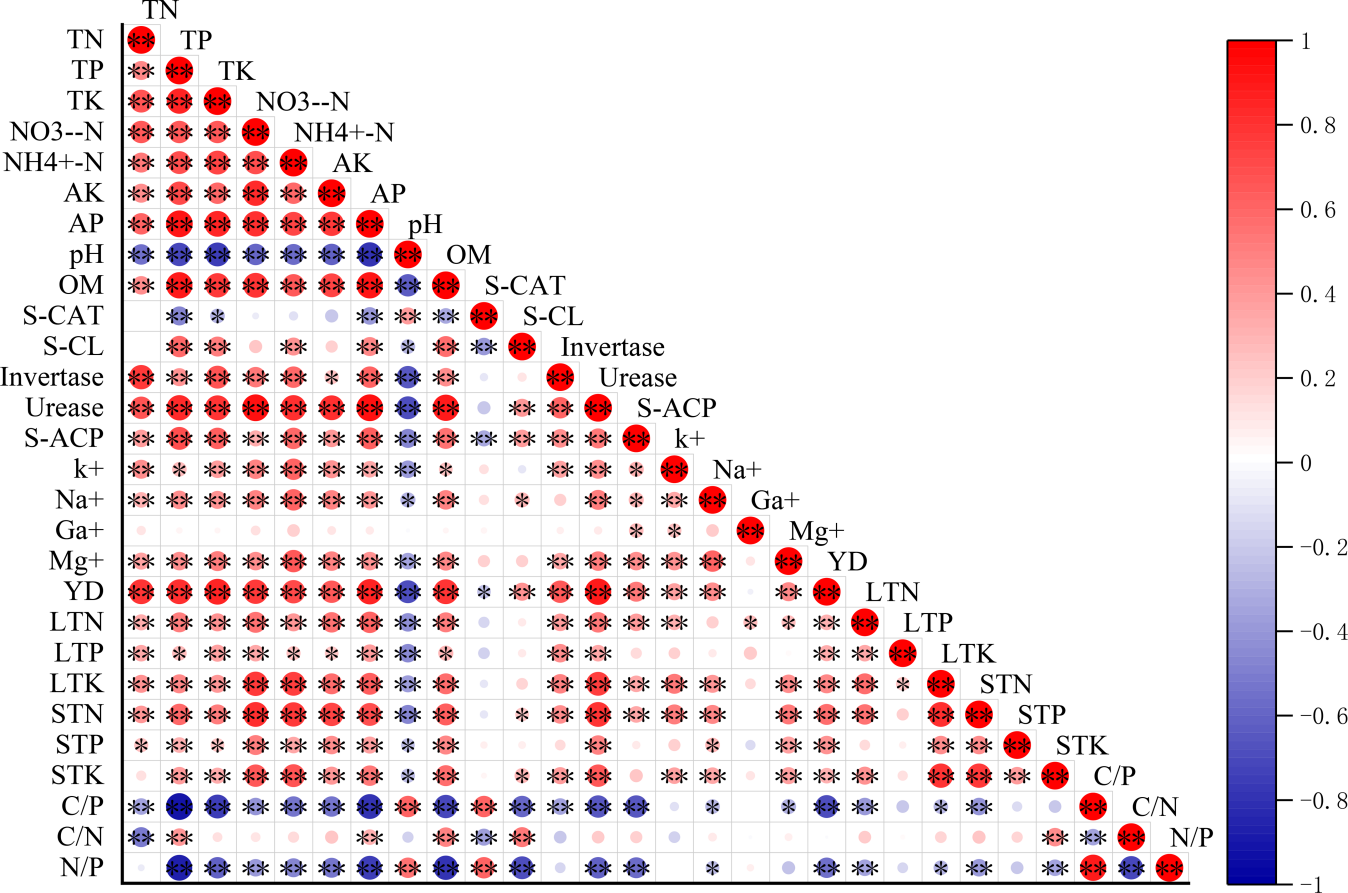
Heatmap of the correlation analysis.

Fertilization regimes significantly influenced soil enzyme activities. The high nitrogen and medium potassium treatment (N_3_K_1_) yielded the highest catalase activity (0.490 mL g^-1^), exceeding the blank (N_0_K_0_) and conventional (N_r_K_r_) controls by 64.59% and 56.36%, respectively (**Figure 2a**). Cellulase activity reached peak (0.055 mg g^-1^) under the medium nitrogen and medium potassium (N_2_K_1_) conditions, whereas the high nitrogen and high potassium treatment (N_3_K_2_) simultaneously enhanced both invertase (3.06 mg g^-1^) and urease (0.98 mg g^-1^) activities, corresponding to 75.56% and 6.46-fold increases than the control (**Figures 2b, c**). Acid phosphatase activity demonstrated equivalent elevation (2.20 mg g^-1^) in the medium nitrogen and medium potassium (N_2_K_1_), and high nitrogen and high potassium treatments (N_3_K_2_), which was significantly higher than other treatments (*P* < 0.05). Correlation analysis showed that urease was significantly positively correlated with total nitrogen (r = 0.89, *P* < 0.01) and nitrate nitrogen (r = 0.78, *P* < 0.01) (**Figure 3**).

### 3.2 Effects of different treatments on sugarcane yield and nutrient uptake

**Figure 4:**
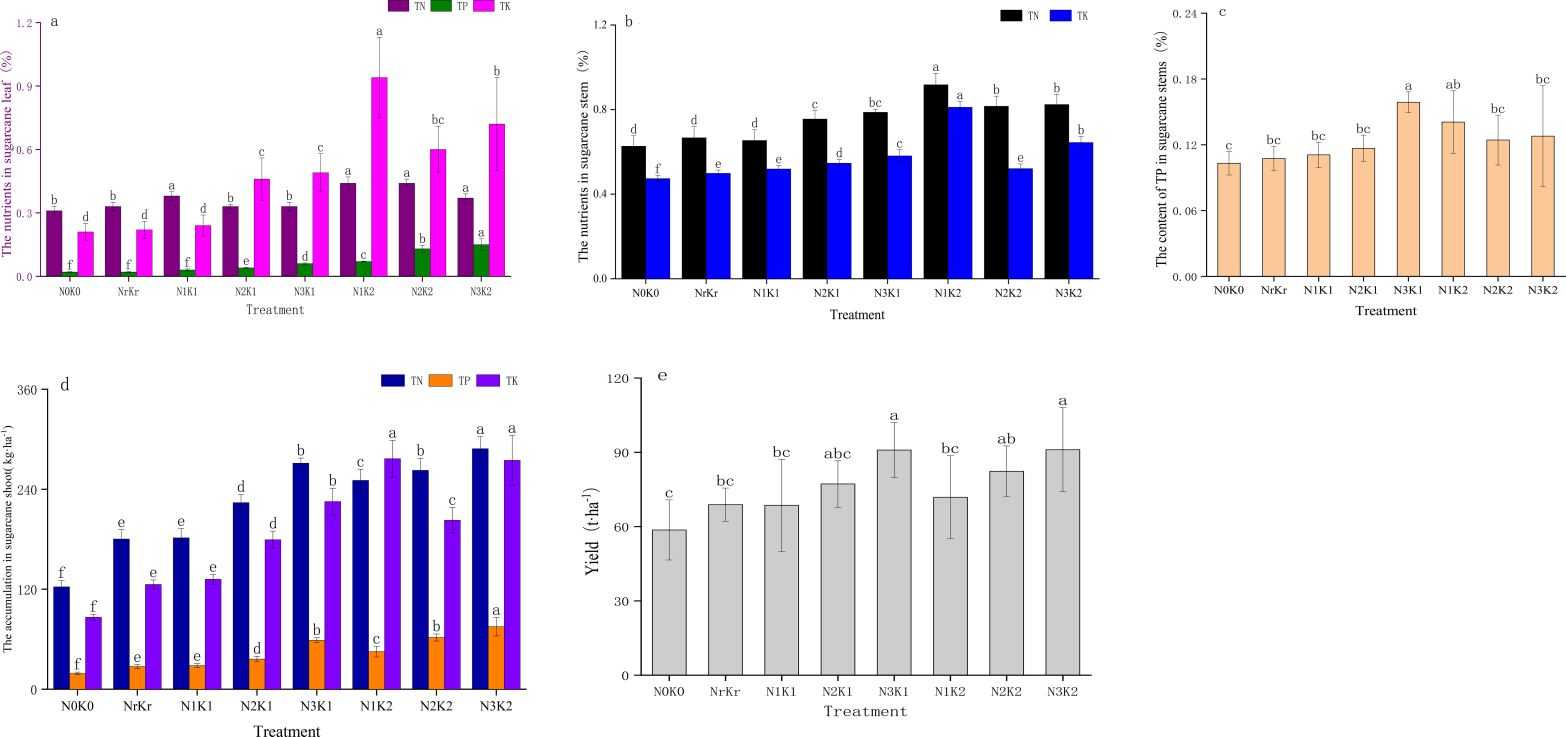
Effect of different treatments on the nutrient content and yield of sugarcane (mean ± SD, n = 3): TN, TP and TK of leaf **(a)**; TN and TK of stem **(b)**; TP of stem **(c)**; TN, TP and TK of accumulation **(d)**; yield **(e)**.

The contents of total nitrogen, total phosphorus and total potassium in sugarcane leaves reached the peak (0.99%, 0.59% and 1.89%) in the treatments of medium nitrogen and high potassium (N_2_K_2_), high nitrogen and high potassium (N_3_K_2_) and low nitrogen and high potassium (N_1_K_2_), respectively (**Figure 4a**). The low nitrogen and high potassium treatment (N_1_K_2_) produced medium stem concentrations of total nitrogen (0.92%) and total potassium (0.81%), whereas the high nitrogen and medium potassium treatment (N_3_K_1_) achieved optimal total phosphorus content (0.16%) (**Figures 4b, c**). Shoot accumulation of nitrogen and phosphorus peaked under the treatment of high nitrogen and high potassium (N_3_K_2_) conditions at 288.65kg ha^-1^ and 75.03kg ha^-1^, respectively (**Figure 4d**). The accumulation of potassium was the largest in the treatment of low nitrogen and high potassium (N_1_K_2_), (**Figure 4e**) reaching 276.66kg ha^-1^. The yield results showed that the high nitrogen and high potassium treatment (N_3_K_2_) had the highest yield (91.05 t ha^-1^), increasing by 55.25% and 32.21% compared with the control and conventional fertilization, respectively (**Figure 4e**). Although the low nitrogen and high potassium treatment (N_1_K_2_) showed lower yield (71.85 t ha^-1^), it demonstrated superior nitrogen use efficiency (38.47%), representing a 21.41% improvement over conventional fertilization (**Table 3**). Strong positive correlations emerged between yield and both total nitrogen (r = 0.85, *P* < 0.01) and total potassium (r = 0.89, *P* < 0.01) (**Figure 3**).

### 3.3 Effects of different treatments on base ions and ecological stoichiometry

**Figure 5:**
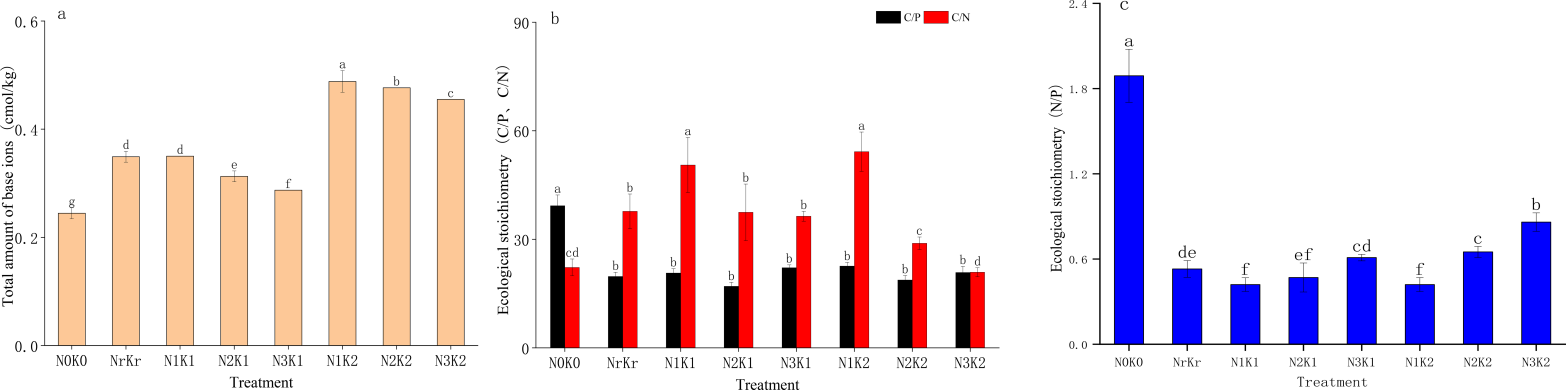
Effects of different treatments on total amount of base ions (mean ± SD, n = 3): **(a)**, ecological stoichiometry of C/P,C/N **(b)**, and ecological stoichiometry of N/P **(c)** in sugarcane soil.

The total amount of base ions in the low nitrogen and high potassium treatment (N_1_K_2_) was the highest (0.49 Cmol kg^-1^), which was significantly positively correlated with total potassium (r = 0.79, *P* < 0.01) and available potassium (r = 0.75, *P* < 0.01) (**Figure 5a**). The treatment of high nitrogen and high potassium (N_3_K_2_) showed the minimal carbon-to-nitrogen ratio (RCN = 20.91), though still exceeding the national average (11.9), indicating reduced organic matter decomposition rates. Carbon-to-phosphorus ratios (RCP) varied substantially. Control plots displaying the highest values (39.28) and the medium nitrogen and high potassium treatment (N_2_K_2_) achieving the lowest levels (17.00), which was lower than the national average (61), reflecting enhanced phosphorus utilization efficiency (**Figure 5b**). Nitrogen-to-phosphorus ratios (RNP) peaked in the control treatment (1.89), remaining markedly below the national threshold (10), confirming prevalent nitrogen limitation throughout the study area (**Figure 5c**).

### 3.4 Soil quality evaluation

Principal component analysis of nine soil fertility indexes (total nitrogen, nitrate nitrogen, ammonium nitrogen, total phosphorus, available phosphorus, total potassium, available potassium, organic matter, and pH) yielded a single significant component (**Table 3**), with the Kaiser-Meyer-Olkin measure verifying sampling adequacy (KMO = 0.821). This component accounted for 72.997% of the total variance. According to the results of principal component analysis of soil quality indicators (**Table 4**), Principal component 1 (72.997%) had the highest loads of total phosphorus (0.907), organic matter (0.902) and available phosphorus (0.969), reflecting nutrient availability. Soil quality assessment revealed significant treatment effects, with the high nitrogen and high potassium treatment (N_3_K_2_) demonstrating optimal performance (47.72), closely followed by the medium nitrogen and high potassium treatment (N_2_K_2_, 46.92). The unfertilized control treatment exhibited markedly inferior results (-2.38).

**Table 4:** Principal component analysis.

IND	TN	TP	TK	NO- 3-N	NH+ 4-N	AK	AP	pH	OM	Eigenvalue	CR (%)	ACR (%)
Fraction1	0.686	0.907	0.895	0.868	0.805	0.82	0.969	-0.807	0.902	6.57	72.997	72.997

Analysis of soil comprehensive fertility index across fertilization treatments (**Table 5**) revealed treatment 7 (N_2_K_2_) achieved the highest index values (0.57), significantly exceeding conventional fertilization (N_r_K_r_, 0.53) and reaching the medium fertility level. The unfertilized control treatment (N_0_K_0_) recorded the lowest values (0.34), demonstrating the efficacy of optimized nutrient management in enhancing soil fertility. Significant positive correlations emerged between IFI and both total nitrogen (r = 0.78, *P* < 0.01) and base ions contents (r = 0.81, *P* < 0.01) (**Figure 3**).

**Table 5:** Comprehensive score and the scores of the principal components of the soil fertility index.

Treatment	1 (N_0_K_0_)	2 (N_r_K_r_)	3 (N_1_K_1_)	4 (N_2_K_1_)	5 (N_3_K_1_)	6 (N_1_K_2_)	7 (N_2_K_2_)	8 (N_3_K_2_)
Fraction1	-2.38	17.43	28.31	34.25	42.68	44.16	46.92	47.72
CS	-2.38	17.43	28.31	34.25	42.68	44.16	46.92	47.72
Rank	8	7	6	5	4	3	2	1
IFI	0.34	0.53	0.53	0.55	0.54	0.53	0.57	0.56

### 3.5 Multidimensional statistical analysis of soil nutrients, enzyme activity, yield and ecological stoichiometry

Correlation analysis (**Figure 3**) revealed strong positive associations between crop yield and both urease activity (r = 0.83, *P* < 0.01) and total potassium content (r = 0.89, *P* < 0.01) (**Figure 3**). The total amount of base ions exhibited a significant correlation with total potassium (r = 0.79, *P* < 0.01) and available potassium (r = 0.75, *P* < 0.01), confirming its role in promoting nutrient availability. Path analysis identified urease as having the greatest direct influence on yield (1.977), while total potassium exerted substantial indirect effects mediated through urease activity (0.702). The direct effect of nitrate nitrogen on yield was negative (-0.85), but the indirect effect through urease was positive (1.84) (**Table 6**).

**Table 6:** Paths analysis of soil nutrient properties and enzyme activities on yield.

Factor	Direct effect	Indirect effect																	
		→TN	→TP	→TK	→NO- 3-N	→NH+ 4-N	→AK	→AP	→pH	→OM	→k+	→Na+	→Ga+	→Mg+	→S-CAT	→S-CL	→Invertase	→Urease	→S-ACP
TN	0.409		0.19	0.27	0.26	0.21	0.19	0.25	-0.23	0.18	0.19	0.13	0.04	0.15	0.00	0.00	0.32	0.27	0.16
TP	0.016	0.01		0.01	0.01	0.01	0.01	0.01	-0.01	0.01	0.00	0.01	0.00	0.01	-0.01	0.01	0.01	0.01	0.01
TK	0.253	0.17	0.21		0.16	0.18	0.15	0.22	-0.19	0.20	0.10	0.11	0.01	0.13	-0.07	0.13	0.17	0.20	0.16
NO- 3-N	-0.85	-0.55	-0.55	-0.55		-0.59	-0.70	-0.70	0.51	-0.67	-0.42	-0.42	-0.10	-0.38	0.05	-0.19	-0.45	-0.80	-0.28
NH+ 4-N	-0.232	-0.12	-0.16	-0.17	-0.16		-0.14	-0.17	0.13	-0.15	-0.14	-0.14	-0.05	-0.15	0.03	-0.10	-0.14	-0.19	-0.14
AK	0.06	0.03	0.04	0.04	0.05	0.03		0.05	-0.04	0.04	0.03	0.03	0.01	0.03	-0.01	0.01	0.02	0.05	0.02
AP	-0.765	-0.46	-0.70	-0.65	-0.63	-0.56	-0.59		0.60	-0.70	-0.26	-0.31	-0.07	-0.33	0.30	-0.35	-0.47	-0.72	-0.48
pH	-0.106	0.06	0.08	0.08	0.06	0.06	0.07	0.08		0.07	0.04	0.03	0.00	0.04	-0.04	0.03	0.07	0.07	0.05
OM	0.126	0.06	0.11	0.10	0.10	0.08	0.09	0.11	-0.08		0.03	0.06	0.01	0.06	-0.04	0.07	0.06	0.11	0.07
k^+^	-0.023	-0.01	-0.01	-0.01	-0.01	-0.01	-0.01	-0.01	0.01	-0.01		-0.01	-0.01	-0.01	0.00	0.00	-0.01	-0.01	-0.01
Na^+^	-0.025	-0.01	-0.01	-0.01	-0.01	-0.02	-0.01	-0.01	0.01	-0.01	-0.01		-0.01	-0.02	0.00	-0.01	0.00	-0.01	-0.01
Ga^+^	-0.035	0.00	0.00	0.00	0.00	-0.01	0.00	0.00	0.00	0.00	-0.01	-0.01		0.00	0.00	0.00	0.00	0.00	-0.01
Mg^+^	0.01	0.00	0.00	0.01	0.00	0.01	0.00	0.00	0.00	0.00	0.00	0.01	0.00		0.00	0.00	0.00	0.01	0.00
S-CAT	-0.026	0.00	0.01	0.01	0.00	0.00	0.01	0.01	-0.01	0.01	0.00	0.00	0.00	0.00		0.01	0.00	0.01	0.01
S-CL	0.021	0.00	0.01	0.01	0.00	0.01	0.00	0.01	-0.01	0.01	0.00	0.01	0.00	0.00	-0.01		0.00	0.01	0.01
Invertase	-0.089	-0.07	-0.04	-0.06	-0.05	-0.05	-0.02	-0.06	0.06	-0.04	-0.03	-0.02	-0.01	-0.03	0.01	-0.01		-0.05	-0.04
Urease	1.955	1.30	1.62	1.56	1.84	1.60	1.57	1.83	-1.35	1.73	0.90	1.01	0.17	0.99	-0.43	0.82	1.20		1.03
S-ACP	-0.044	-0.02	-0.03	-0.03	-0.01	-0.03	-0.02	-0.03	0.02	-0.02	-0.01	-0.01	-0.01	-0.02	0.01	-0.02	-0.02	-0.02	

The coefficient of determination R^2^ = 0.982, the coefficient of remaining diameter = 0.134.

**Figure 6:**
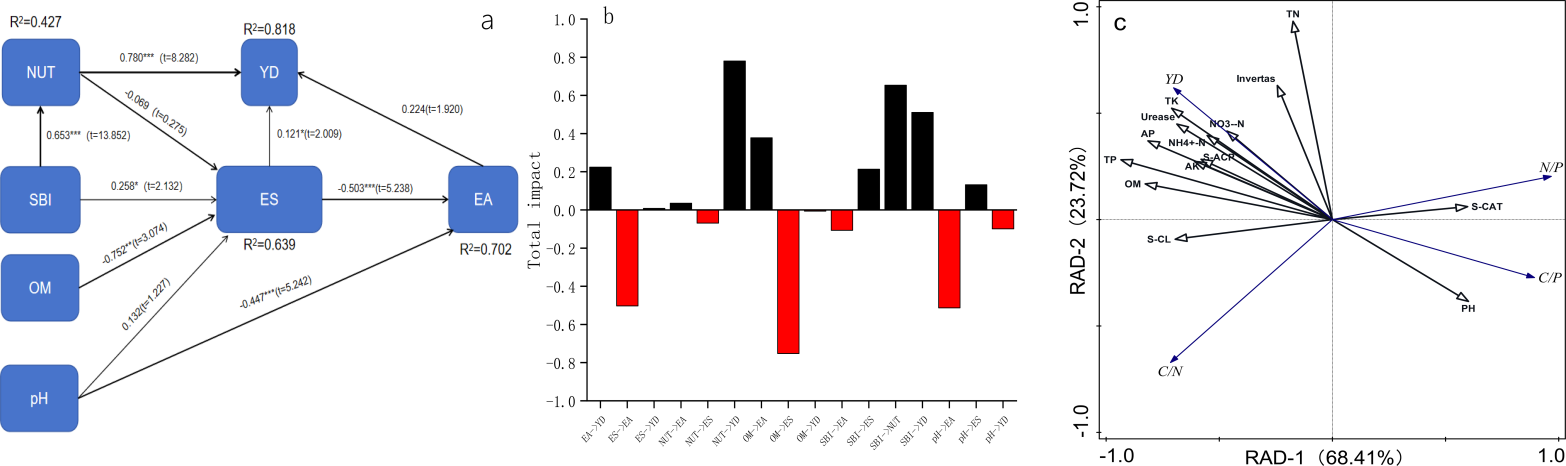
The equation structure model **(a)**, the total effect diagramand **(b)** and redundancy analysis plots **(c)** of ecological stoichiometry, soil nutrient, and enzyme activity.

The structural model showed that the direct effect of soil nutrients on yield (0.780) was dominant, and indirectly affected yield through enzyme activity (0.702) (**Figures 6a, b**). The base ions (exchangeable potassium, sodium, calcium, magnesium) were significantly positively correlated with soil nutrient content (r = 0.803), confirming their fertility-enhancing properties. Principal component analysis (**Figure 6c**) demonstrated that the first axis (68.41%) predominantly reflected positive associations between RCN, catalase activity, and pH, while the second axis (23.72%) captured yield-nutrient relationships. Yield was strongly positively correlated with urease, total phosphorus and available phosphorus, contrasting with the negative association observed with pH. Structural equation and redundancy analysis revealed that available phosphorus led to yield formation through direct supply and promotion of base ions activation, while the high nitrogen and medium potassium treatment (N_3_K_1_) increased nutrient accumulation in a short term, but aggravated nitrate nitrogen accumulation and imbalance of carbon-to-nitrogen ratio.

Grey correlation analysis (**Table 7**) identified total potassium as exhibiting the strongest yield correlation (0.911), followed sequentially by invertase activity, organic matter content, and exchangeable magnesium, all demonstrating coefficients exceeding 0.9. Nitrate nitrogen showed the weakest association with crop yield among the measured parameters.

**Table 7:** Correlation degree and rank order of soil nutrient properties and enzyme activities with yield.

IND	TN	TP	TK	NO- 3-N	NH+ 4-N	AK	AP	pH	OM	k+	Na+	Ga+	Mg+	S-CAT	S-CL	Invertase	Urease	S-ACP
Correlation	0.872	0.881	0.911	0.67	0.779	0.875	0.88	0.866	0.902	0.82	0.83	0.863	0.9	0.836	0.755	0.909	0.833	0.783
Order	8	5	1	18	16	7	6	9	3	14	13	10	4	11	17	2	12	15

## 4 Discussion

### 4.1 Regulation mechanism of nitrogen and potassium coupling on soil nutrient transformation and enzyme activity

This study found that the high nitrogen treatment (N_3_K_2_) significantly elevated urease activity (0.98 mg g^-1^) and nitrate nitrogen content (26.64 mg kg^-1^) while inducing soil acidification (pH 4.57), consistent with reported nitrogen-mediated acidification effects (Liu et al., 2024). Contrastingly, the low nitrogen and high potassium treatment (N_1_K_2_) enhanced nitrogen use efficiency (38.47%) through stimulated urease activity (0.96 mg g^-1^) and invertase activity (2.11 mg g^-1^), supporting the enzyme-mediated nitrogen transformation mechanism (Holatko et al., 2025). Notedly, phosphorus dynamics revealed differential responses: the medium nitrogen and high potassium treatment (N_2_K_2_) accumulated total phosphorus (1.38 g kg^-1^) but limited available phosphorus increment (3.2%), potentially attributable to iron/aluminum oxides fixation in acidic conditions (Wen et al., 2023).

### 4.2 Driving effect of nitrogen and potassium interaction on nutrient uptake and yield formation of sugarcane

The high nitrogen and high potassium treatment (N_3_K_2_) maximized yield (91.05 t ha^-1^) through enhanced nitrogen accumulation (288.65 kg ha^-1^), yet demonstrated limited nitrogen use efficiency (24.95%), which was consistent with the luxury absorption theory proposed by Bronson (2021). Conversely, the low nitrogen and high potassium treatment (N_1_K_2_) elevated the nitrogen use efficiency to 38.47% by optimizing the carbon-nitrogen ratio (RCN = 54.23), corroborating potassium-mediated nitrogen assimilation mechanisms reported by Kumari et al. (2023). Path analysis showed that the direct effect of urease on yield (1.977) was significantly higher than other indicators. Grey correlation analysis confirmed the predominant influence of total potassium on yield (r = 0.911), corroborating the potassium-mediated root development-nutrient acquisition regulatory mechanism described by Nabati et al. (2025).

### 4.3 Multi-dimensional response characteristics of soil quality evolution

Principal component analysis revealed optimal soil fertility (IFI = 0.57) under the medium nitrogen and high potassium treatment (N_2_K_2_), concurrent with elevated total amount of base ions (0.48 Cmol/kg) surpassing other treatments. These findings support the established base ion-fertility correlation documented by Ordoñez et al. 2010. The ecological stoichiometric characteristics showed that the soil in the study area is in the states of nitrogen limitation (RNP = 0.42-1.89) and phosphorus sufficiency (RCP = 17.00-22.60), which was lower than the RNP threshold theory proposed by Koerselman and Meuleman (1996). Elevated tropical temperatures and humidity in India likely enhance microbial activity, resulting in accelerated nitrogen mineralization rates (higher RNP values). In this study, observed RNP values remained substantially lower (8.2-12.5) than those reported for Brazilian sugarcane regions, characteristic of nitrogen-limited karst soils (Batjes, 1996). Similarly, reduced RCP values (17.00-22.60 compared to 48–62 in Florida) reflect pronounced phosphorus fixation in karst soils (as mentioned in section 3.1 of the document for acidic soil phosphorus fixation), but long-term excessive phosphorus application by farmers (such as 3.13 kg acre^-1^ of superphosphate) improved effectiveness. Structural equation models revealed soil nutrient mediation of yield through enzymatic activity (total effect = 0.702), confirming the elemental coupling hypothesis proposed by Reiners (1986). It was worth noting that the carbon-nitrogen ratio imbalance (RCN = 20.91) caused by high nitrogen treatment was significantly higher than the global average (11.9), suggesting organic matter decomposition inhibition, aligning with Cleveland and Liptzin (2007) regarding RCN-mediated microbial community modulation. These findings offer region-specific implications for nutrient management in tropical/subtropical agricultural ecosystems.

### 4.4 Sustainability trade-off of nitrogen-potassium ratio optimization

The optimized N:K_2_O ≈ 1:1.5 fertilization regime demonstrated dual benefits of sustaining sugarcane yield (82.35 t ha^-1^) while preserving soil fertility (IFI = 0.57). This balanced approach maintained ammonium nitrogen activation above 1.5% and RCP below 25, effectively nutrient efficiency with environmental protection. Compared with the conventional recommendations (Delfim et al., 2025), the proposed scheme exhibited 21.41% greater nitrogen use efficiency and superior acidification mitigation (ΔpH = +0.34). However, exceeding 2.3% ammonium nitrogen activation in Zhanjiang, Guangdong, lateritic soils triggered notable nitrate accumulation risks (Xu et al., 2010). The activation coefficient of paddy soil in the Taihu Lake Lake basin > 0.8% is accompanied by nitrogen loss (Yang et al., 2004). In karst sugarcane areas, the 1.5% ammonium nitrogen activation threshold serves as a reliable environmental risk indicator, corresponding to nitrate accumulation below 50 mg kg^-1^ (N_2_K_2_: 45.6 mg kg^-1^; **Figure 1b**) while maintaining strong yield-ammonium nitrogen correlation (r = 0.9) (**Table 6**), thereby optimizing nutrient use efficiency. However, long-term high potassium input may lead to soil potassium accumulation (total potassium 16.44 g kg^-1^). It was necessary to establish an accurate regulation model based on soil potassium activation coefficient in combination with the dynamic management strategy of potassium fertilizer proposed by Mahmood and Ahmed (2024). The proposed N:K_2_O ≈ 1:1.5 in this study fertilization regime enhances both yield and potassium use efficiency during single growing seasons, but requires long-term evaluation due to inherent karst soil characteristics. Potassium fixation in such soils creates disparity between accumulation and effectiveness, while nitrogen nitrification and potassium anion loss pose a risk of exacerbating soil acidification. Implementation of long-term field trials incorporating crop rotation optimization and strategic lime application is recommended to achieve sustainable yield-soil health equilibrium.

## 5 Conclusion

This investigation employed field positioning experiments and multidisciplinary methods to elucidate nitrogen-potassium coupling mechanisms governing sugarcane productivity and soil quality in karst ecosystems. The high nitrogen and high potassium treatment (N_3_K_2_) achieved the highest yield (91.05 t ha^-1^) while inducing soil acidification. In contrast, the low nitrogen and high potassium treatment (N_1_K_2_) demonstrated superior nitrogen use efficiency (38.47%, 18.41% above conventional practice) and established an optimized nitrogen transformation pathway, despite yielding comparatively less. Path analysis and grey correlation analysis showed that urease and total potassium played a dominant role in the direct effect and correlation degree of yield respectively. The N_2_K_2_ treatment demonstrated optimal soil quality enhancement, significantly elevating total nitrogen, total potassium, and base ions while achieving peak integrated fertility index (IFI = 0.57). The ecological stoichiometry analysis revealed nitrogen-limited but phosphorus-efficient soil conditions, with N_2_K_2_ treatment effectively stabilizing nutrient cycle. For southern sugarcane cultivation, implementation of N:K_2_O ≈ 1:1.5 fertilization is advised, utilizing ammonium nitrogen activation coefficient (> 1.5%) and RCP (< 25) as primary fertility monitoring parameters, thereby establishing a scientific framework for sugarcane precision fertilization and soil sustainable management in karst areas. To validate conclusions generalizability, multi-regional experiments across diverse sugarcane cultivation zones (Nanning and Chongzuo) will examine influences of soil types, while multi-genotype comparisons (Guitang 55, Yuetang 93-159) will assess genetic influences on nitrogen and potassium response. Future investigations should integrate long-term field experiments with economic analyses to optimize region-specific nitrogen-potassium coupling strategies.

## Data Availability

The original contributions presented in the study are included in the article/supplementary material. Further inquiries can be directed to the corresponding authors.
